# How is mental health associated with teenagers’ attitudes toward sport participation? — The chain mediating role of exercise self-efficacy and sport confidence

**DOI:** 10.3389/fpsyg.2026.1892031

**Published:** 2026-07-13

**Authors:** Jie Qiu, Mengfen Liu, Guiyun Zhang, Jiawei Chen, Haoyan Huang

**Affiliations:** 1 Hunan Mechanical and Electrical Polytechnic, Changsha, China; 2 Faculty of Educational Sciences, University of Helsinki, Helsinki, Finland

**Keywords:** exercise self-efficacy, mental health, sport confidence, sport participation attitudes, teenagers

## Abstract

**Purpose:**

To elucidate the intrinsic psychological dynamics through which Mental Health is associated with Sport Participation Attitudes in adolescents, this study examines its direct and indirect effects through Exercise Self-Efficacy and Sport Confidence, developing a chained mediation model.

**Methods:**

A stratified cluster sampling method was employed to select 1,384 adolescents aged 12–18 from Hunan Province as research subjects. A validated questionnaire was administered, and data were analyzed using SPSS and the PROCESS macro.

**Results:**

Adolescent mental health has a significant positive association with attitudes toward sport participation, and exercise self-efficacy and sport confidence partly explain its effects as mediators. A significant chain mediation path is established between exercise self-efficacy and sport confidence, suggesting that mental health is associated with adolescents’ sport participation attitudes through improving exercise self-efficacy and subsequently strengthening sport confidence.

**Conclusion:**

This study clarifies the hierarchical psychological transmission mechanisms by which mental health is associated with adolescents’ sport participation attitudes, and identifies the progressive relationships of cognitive belief variables. These findings provide empirical evidence for adolescent mental health education, the optimization of campus sports teaching, and interventions in sport participation attitudes.

## 1 Introduction

Mental health serves as a fundamental factor for the healthy growth and comprehensive development of adolescents. Adolescents are in a critical period marked by rapid development in emotional regulation and stress resilience, yet remain immature (Silvers, 2022). Under the cumulative influence of academic and societal pressure, they are inclined to become susceptible to psychological distress. Empirical surveys indicate that the overall prevalence of mental disorders among adolescents in China reaches 8.9% (Dong et al., 2025). Mental health issues have emerged as a significant public health concern that constrains adolescent development.

The decreasing mental health issue has brought substantial negative impacts to various wellbeing and flourishing outcomes, with sport participation attitudes receiving increasing concerns. A positive attitude toward physical activity not only serves as a significant predictor of higher levels of physical activity participation among adolescents, but also acts as a protective factor against depression, anxiety and academic stress (Nelson et al., 2010; Park and Kim, 2008; Rullestad et al., 2021). The physical activity-related beliefs and behavioral habits formed during adolescence often have long-lasting effects that extend into adulthood (Loprinzi et al., 2012); fostering a positive attitude toward physical activity among this group should therefore be regarded as a key early intervention strategy for promoting mental health in young people. However, the underlying dynamics of how mental health is associated with sport participation attitudes remain insufficiently elucidated. To further the understanding of this dynamic, exercise cognitive psychological factors can emerge as the core mediating pathway linking individual psychological states with sporting behavior (Gill et al., 2017). Among these, exercise self-efficacy and sport confidence are key traits determining adolescents’ willingness to engage in sports, as well as the consistency and depth of their participation (Feltz et al., 2008; McGrane et al., 2016). A positive mental health state facilitates adolescents in building affirmative self-perceptions, enhancing their exercise efficacy and confidence levels, thereby promoting active participation in physical activities. Conversely, unhealthy psychological conditions weaken exercise cognitive beliefs and suppress sport participation attitudes, finally resulting in weaker sport participation attitudes. Existing studies on youth physical activity and sport psychology have yielded fruitful results. Buyrukoğlu et al. (2024a,b) analyzed the link between digital game addiction and outdoor play of young children, while clarified the psychological and social effects of indoor and outdoor sports on preschoolers. Buyrukoğlu (2023) focused on children’s sports imagery, which is closely related to sport confidence. In addition, a meta-analysis by Kayantaş et al. (2022) revealed the influence of family factors on physical activity. These cross-cultural findings effectively support the theoretical basis of the current chain mediation model and help comprehensively interpret the relationships between mental health, exercise self-efficacy and adolescents’ sport participation attitudes.

Based on this, the present study aimed to examined the role of mental health on sport participation attitudes. It introduces exercise self-efficacy and sport confidence to construct a chain mediation model, further exploring their mediating dynamics. These examinations can help validate the theoretical framework of adolescent sports behavioral development and mental-physical coordination, providing empirical support and practical references for empowering adolescent sport participation attitudes through mental health.

### 1.1 The relationship between mental health and sport participation attitudes

Mental Health, as a comprehensive representation of emotional regulation, social adaptation, cognitive functioning, and personality development, is an important antecedent driving various wellbeing and flourishing outcomes (Bijkerk et al., 2023, 2024). From the perspective of health behavior psychology, the initiation and sustained engagement in individual behaviors heavily rely on internal psychological resources rather than being solely driven by external environments. Adolescence is a critical period for the formation of behavioral habits, where stable and positive mental health serves as a fundamental psychological prerequisite for adolescents’ engagement in health behaviors (Wu et al., 2025). Empirical studies have shown that psychological distress, such as anxiety and depression, depletes self-regulatory resources, induces behavioral avoidance, and reduces the frequency, intensity, and sustainability of sport participation attitudes (Chen et al., 2026; Zhu et al., 2026); conversely, adolescents with higher levels of mental health demonstrate stronger emotional regulation capabilities, perceive fewer participation barriers, and are more likely to develop sustained sport participation attitudes (Li et al., 2024; Martinez et al., 2024). Based on this, Hypothesis 1 is proposed: mental health has a significant positive effect on adolescents’ sport participation attitudes.

### 1.2 The mediating role of exercise self-efficacy

To understand how mental health is associated with sport participation attitudes, this study further explored the role of exercise self-efficacy. According to Bandura and Wessels’s (1997) Self-Efficacy Theory, the initiation, engagement, and persistence of individual behavior are regulated by self-perceptions of competence in specific domains. Exercise self-efficacy refers to an individual’s judgment and subjective belief regarding their ability to complete exercise tasks, overcome difficulties, and persist in their training, serving as a proximal core factor in predicting exercise behavior (Feltz et al., 2008). Therefore, exercise self-efficacy is a critical antecedent of sport participation attitudes. Adolescents with high self-efficacy are more likely to face sports challenges positively, engage actively, and maintain their exercise habits; conversely, those with low self-efficacy are prone to avoidance and withdrawal, inhibiting engagement behaviors (Babic et al., 2014; Grasaas and Sandbakk, 2024; Hamilton et al., 2017).

On the other hand, Good mental health serves as an important reserve of psychological resources. Adolescents with high mental health levels exhibit low reactivity to negative emotions and strong coping abilities under stress, fostering positive self-perceptions and consequently enhancing their exercise self-efficacy. In contrast, psychological distress depletes psychological resources, leading to negative perceptions of exercise ability and a diminished sense of self-efficacy. Existing studies have confirmed that mental health positively predicts task-specific self-efficacy and optimizes exercise cognition (Lin et al., 2024; Peng et al., 2025). In summary, mental health may be indirectly associated with sport participation attitudes by its effect on exercise self-efficacy. Based on this, Hypothesis 2 is proposed: exercise self-efficacy acts as a mediating factor between mental health and sport participation attitudes among adolescents.

### 1.3 The mediating role of sport confidence

The promotion of mental health on sport participation attitudes can also be achieved through the mediating role of sport confidence. Sport confidence, distinct from cognitively oriented self-efficacy, represents a stable, holistic positive belief and emotional tendency formed within sports contexts. It reflects the degree of certainty individuals have regarding their sports performance, situational adaptation, and challenge response, serving as a crucial emotional resource that drives sustained participation (Budnik-Przybylska et al., 2022; Lochbaum et al., 2022). Compared to cognitive judgments of ability (including exercise self-efficacy), sport confidence places greater emphasis on the positive belief output at the behavioral level, which can shorten the psychological distance between individuals and sporting behaviors, while mitigating tendencies to avoid physical activities (Sniehotta et al., 2005; Weinberg and Gould, 2023). Research has confirmed that sport confidence is a stable proximal predictor of sport participation attitudes (Fernández-Bustos et al., 2026; Nothnagle and Knoester, 2025). Adolescents with high sport confidence harbor more positive attitudes and intentions toward physical activities, enabling them to proactively overcome barriers to participation and enhance the frequency, duration, and persistence of sport participation attitudes. Conversely, insufficient sport confidence may reduce both the level of engagement and the depth of participation in sports (Tang et al., 2025; Zhang et al., 2022).

Moreover, mental health is a fundamental factor for adolescents to build high levels of sport confidence. Adolescents with high levels of mental health tend to be emotionally stable, resilient to setbacks, and possess abundant positive psychological resources. They are likely to develop optimistic expectations for sports, boldly try new activities, face challenges head-on, and maintain stable confidence. In contrast, adolescents experiencing prolonged psychological distress and emotional repression are prone to negative self-suggestions, leading to lower expectations for sports, a retreatist mindset toward challenges, and diminished self-confidence (Murphy et al., 2022; Rosenvinge et al., 2018). Accordingly, we propose Hypothesis 3: sport confidence serves as a mediating factor between mental health and adolescent sport participation attitudes.

### 1.4 The relationship between exercise self-efficacy and sport confidence: a chain mediation dynamic

From the perspective of the internal hierarchical logic of sports psychology, the dynamics in the role of mental health in sport participation attitudes may involve a progressive, sequential chain of pathways. According to the theory of self-efficacy (Kayantaş et al., 2022), stable mental health first shapes exercise self-efficacy; high levels of self-efficacy further internalize into positive beliefs within the context of sports (sport confidence). Specifically, previous research has confirmed that exercise self-efficacy is a precursor cognitive condition for the development of confidence. Positive evaluations of ability can strengthen positive beliefs about sports and facilitate the hierarchical upgrade from cognitive to emotional beliefs (Feltz et al., 2008; Yang and Mojtahe, 2023); a positive psychological state may indirectly influence health behaviors by sequentially enhancing sports cognition and beliefs. The chain mediation model is supported by both theoretical and empirical evidence (Chen et al., 2025; Shen et al., 2026). Therefore, hypothesis 4 is proposed: exercise self-efficacy and sport confidence play a chain mediation role between mental health and adolescents’ sport participation attitudes.

**FIGURE 1:**
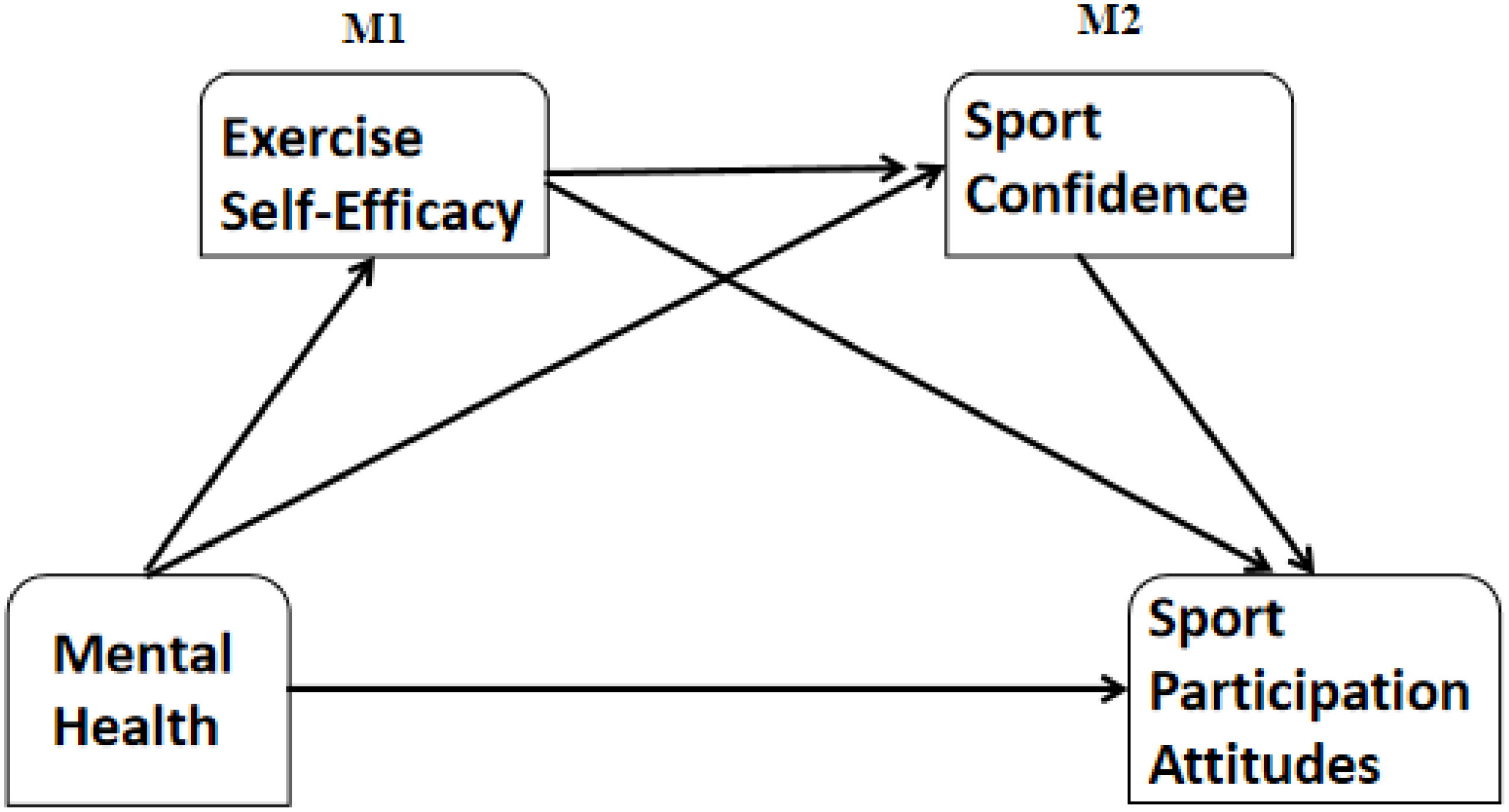
Mediation effect hypothesis model of research framework.

In summary, a robust intrinsic relationship is present among mental health, exercise self-efficacy, sport confidence, and sport participation attitudes; however, existing research often focuses on binary relationships, lacking an integrative analysis of multivariable progression mechanisms. This study utilizes self-efficacy theory as a framework to systematically elucidate the effect of mental health on sport participation attitudes, revealing the independent mediation and chain transmission pathways of the two types of variables to clarify the underlying psychological mechanisms (see Figure 1).

## 2 Materials and methods

### 2.1 Participants and procedures

From September to December 2025, a stratified cluster sampling method was employed, and online questionnaires were distributed and collected via the Wenjuanxing platform (a mainstream professional online questionnaire platform widely used in China for academic research, social surveys and data collection. It supports anonymous questionnaire distribution, automatic data sorting and preliminary data screening). A total of adolescent students from five secondary schools in Hunan Province were recruited as research subjects. Participants were required to meet the following inclusion criteria: aged between 12 and 18 years, capable of independently completing all questionnaire items, possessing normal cognitive function, and having no serious physical illnesses or mental disorders that affect daily physical activities. Before the formal investigation commenced, the research purpose, principles of anonymous data usage, and voluntary participation were fully explained to all participants and their guardians, and written informed consent was obtained prior to data collection. The research protocol was approved by the Ethics Committee of Hunan Mechanical and Electrical Polytechnic (Approval No. 20251209) and strictly adhered to the ethical guidelines of the Declaration of Helsinki. The questionnaire was designed to be completed in approximately 15 min and included three attention check questions to eliminate invalid responses. Participants who failed to complete the questionnaire or submitted invalid responses were excluded from the final statistical analysis. After the questionnaires were collected online by the class teachers, data were exported and cross-verified by two independent researchers. A total of 1,407 questionnaires were collected, and after eliminating invalid and incomplete responses based on attention check questions and logical verification, 1,384 valid samples were retained, resulting in a response rate of 98.36%, with 701 males (50.65%) and 683 females (49.34%).

### 2.2 Measuring tools

#### 2.2.1 Mental health

Adolescents’ mental health status was assessed using the mental health Continuum Short Form (MHCSF). This scale was originally developed by Keyes et al. (2008) and later revised into Chinese by Yin and He (2012). The MHCSF includes 14 items that cover three core dimensions: emotional wellbeing, social wellbeing, and mental health. All items were rated on a six-point Likert scale, ranging from 1 (never) to 6 (every day). Higher total scores indicate more positive mental health status. The single-factor model showed adequate fit (χ^2^/df = 17.509, CFI = 0.92, TLI = 0.92, SRMR = 0.08). In this study, the Cronbach’s α coefficients for the total score, emotional wellbeing, social wellbeing, and mental health were 0.845, 0.790, 0.698, and 0.844, respectively, indicating satisfactory internal consistency.

#### 2.2.2 Sport participation attitudes

The Sport Participation Attitudes scale developed by Zhi et al. (2023), was adopted in this study. The original scale was designed specifically for tennis participants. To fit the research context focusing on general adolescent physical activity, we conducted minor item adaptation: we merely replaced the term tennis with physical activities across all six items. No adjustments were made to item content, sentence structure, response options or item quantity, so the core meaning of each item remained unchanged. To guarantee content validity and linguistic appropriateness for adolescent groups, five interdisciplinary experts (two sport psychology researchers, two senior physical education teachers and one adolescent development scholar) were invited to review the revised items. All experts agreed that the revised wording was accurate, the item connotation was consistent with the original version, and the scale was suitable for the target population. Prior to formal data collection, a pilot test was carried out among 156 adolescents with similar backgrounds. The Cronbach’s α coefficient of the revised scale in the pilot sample was 0.926, indicating good internal consistency. Exploratory factor analysis confirmed that the scale retained its original unidimensional factor structure. In the formal sample, the scale adopted a five-point Likert rating. Higher scores represent stronger intrinsic motivation and more positive attitudes toward physical activity participation. Confirmatory factor analysis was further performed to verify the factor structure of the revised scale. The single-factor model showed adequate fit (χ^2^/df = 31.621, CFI = 0.96, TLI = 0.933, SRMR = 0.040). The Cronbach’s α of the scale in the formal study was 0.929, demonstrating stable and satisfactory psychometric properties after revision.

#### 2.2.3 Exercise self-efficacy

Exercise self-efficacy is measured by revised exercise self-efficacy Scale from Sun et al. (2005). This scale contains a total of 10 items and employs a six-point Likert scale for scoring, ranging from “strongly disagree” to “strongly agree,” with scores ranging from 1 to 6. Items 2, 4, 5, 6, and 7 require reverse scoring. A higher total score on the scale indicates a stronger level of exercise self-efficacy. The single-factor model showed adequate fit (χ^2^/df = 76.10, CFI = 0.9, TLI = 0.93, SRMR = 0.061). In this study, the Cronbach’s α coefficient for the scale was 0.851.

#### 2.2.4 Sport confidence

The Sport Confidence scale developed by Shen (2004) was used in this study. The original scale was designed for sports competition contexts. Considering that our research focuses on adolescents’ daily physical exercise rather than formal competitions, we implemented targeted contextual adaptation following international scale adaptation guidelines. Among the total eight items of the scale, eight items involving competition-related descriptions were slightly revised: we replaced words such as competition, match, contest with general expressions of daily exercise, physical activity, respectively. Importantly, we did not modify the core connotation, sentence structure, Likert response format and scoring rules of all items, so the original measurement intention was fully retained. To ensure content and linguistic validity, five experts were invited to evaluate the revised items, including two sport psychology researchers, two senior sports coaches and one adolescent physical education specialist. All experts agreed that the contextual revision was reasonable, the item semantics remained consistent with the original scale, and the expressions were suitable for adolescent exercise scenarios. A pilot test was conducted on 156 adolescents before formal data collection. The Cronbach’s α of the adapted scale in the pilot sample was 0.783. Exploratory factor analysis verified that the scale still maintained its original unidimensional structure. In the formal sample, all items adopted a six-point Likert scale. Higher scores indicate higher levels of sport confidence. Confirmatory factor analysis was conducted to verify the factor structure of the adapted scale. The single-factor model achieved good fit (χ^2^/df = 15.898, CFI = 0.92, TLI = 0.9, SRMR = 0.062). The Cronbach’s α coefficient was 0.790. All indicators demonstrate that the adapted scale has stable reliability and satisfactory construct validity.

To comprehensively evaluate the psychometric properties of all scales among the adolescent sample, confirmatory factor analysis (CFA) was performed. The overall fit indices of the four-factor measurement model were acceptable. We further calculated composite reliability (CR) and average variance extracted (AVE) to assess convergent validity. As shown in Table 1, all CR values were greater than 0.7 and all AVE values exceeded 0.5, demonstrating good composite reliability and convergent validity. Discriminant validity was verified by comparing the square root of AVE of each construct with inter-construct correlations (see Table 1): the square root of AVE for each latent variable was larger than its correlation coefficients with other constructs, indicating satisfactory discriminant validity.

**TABLE 1:** Reliability and convergent validity of latent variables (*N* = 1,384).

Latent variable	Number of items	Cronbach’s α	CR	AVE
Mental health	14	0.85	0.86	0.55
Exercise self-efficacy	10	0.85	0.86	0.54
Sport confidence	8	0.79	0.82	0.59
Sport participation attitudes	6	0.93	0.93	0.69

Cut-off criteria, Cronbach’s α > 0.7, CR > 0.7, AVE > 0.5.

### 2.3 Statistical analysis

Descriptive statistics and correlation analysis were conducted using SPSS version 26.0, while the PROCESS macro was employed to test the mediation effects. Given that all data were collected via self-reported questionnaires, two strategies were adopted to assess potential common method bias (CMB). First, Harman’s single-factor test was conducted. Thirteen factors with eigenvalues greater than 1 were extracted, and the first unrotated factor explained 21.483% of the total variance, which was far lower than the 40% critical value. Second, we conducted a competing model test based on confirmatory factor analysis (see Table 2). We compared the fit of the hypothesized four-factor model and a single-factor model where all observed items were loaded on one general factor. The four-factor model exhibited excellent model fit, while the single-factor model showed poor fit. The substantial difference in model fit demonstrated that CMB did not seriously bias the present results. Since no dedicated marker items were designed in the questionnaire, the marker variable procedure was not implemented (Podsakoff et al., 2003).

**TABLE 2:** Competing model test (*N* = 1,384).

Model	χ^2^	df	χ^2^/df	CFI	TLI	RMSEA	SRMR
Four-factor model	5621.53	659	8.53	0.92	0.9	0.07	0.07
Single-factor mode	19160.42	665	28.813	0.33	0.29	0.14	0.15

## 3 Research results

### 3.1 Descriptive statistics and correlation analysis

The means, standard deviations, and Pearson correlation matrix for all variables are presented in Table 3. The results indicate that mental health, exercise self-efficacy, sport confidence, and sport participation attitudes exhibit significant positive correlations with each other. Specifically, mental health is significantly positively correlated with exercise self-efficacy, sport confidence, and sport participation attitudes; exercise self-efficacy shows significant positive correlations with sport confidence and sport participation attitudes; and sport confidence is significantly positively correlated with sport participation attitudes. These correlation patterns provide preliminary support for the theoretical hypotheses, establishing a foundation for mediating effect testing.

**TABLE 3:** Descriptive statistics and correlation analysis (*N* = 1,384).

Variable	*M*	SD	MH	ESE	SC	SPA
MH	61.72	10.78	1	–	–	–
ESE	44.38	8.41	0.22***	1	–	–
SC	26.46	5.65	0.14***	0.16***	1	–
SPA	22.01	5.25	0.22***	0.29***	0.20***	1

*P < 0.05, **p < 0.01, ***p < 0.001.

### 3.2 Mediating effect analysis

After controlling for demographic variables, the results of the regression analysis (Table 4) indicate that mental health significantly positively predicts sport participation attitudes (β = 0.22, t = 8.27, *p* < 0.001), exercise self-efficacy (β = 0.22, t = 8.51, *p* < 0.001), and sport confidence (β = 0.11, t = 3.86, *p* < 0.001); exercise self-efficacy significantly positively predicts sport confidence (β = 0.14, t = 5.14, *p* < 0.001). When mental health, exercise self-efficacy, and sport confidence were simultaneously included in the regression equation, all three significantly positively predicted sport participation attitudes (β = 0.15, 0.24, 0.14, *p*_*s*_ < 0.001), and the overall model showed good fit (R^2^ = 0.19, F = 68.72, *p* < 0.001). These results validate the main effects of each path and the premise of chain transmission, providing foundational support for subsequent mediation effect testing.

**TABLE 4:** Regression analysis between variables (*N* = 1,384).

Item	SPA		ESE		SC		SPA	
	β	t	β	t	β	t	β	t
Gender	0.05	1.87	0.04	1.63	0.15***	5.82	0.02	0.67
Age	−0.02	−0.86	−0.05	−1.77	−0.02	−0.67	−0.01	−0.33
MH	0.21***	8.12	0.22***	8.37	0.10***	3.60	0.14***	5.55
ESE	–	–	–	–	0.13***	4.91	0.24***	9.14
SC	–	–	–	–	–	–	0.14***	5.36
R ^2^	0.05		0.05		0.06		0.13	
F	24.23***		26.11***		22.01***		41.30***	

*P < 0.05, **p < 0.01, ***p < 0.001.

The results of the chain mediation analysis (Table 5) indicate that the total effect of mental health on sport participation attitudes is significant (Effect = 0.106, 95% CI = [0.081, 0.131]). Additionally, the direct effect is also significant (Effect = 0.070, 95% CI = [0.046, 0.095]), suggesting that mental health has both direct and indirect promoting effects. The total indirect effect is significant (Effect = 0.035, 95% CI = [0.026, 0.045]), and all decomposition paths are also significant.

**TABLE 5:** Chain mediation effect analysis (*N* = 1,384).

Effect	Influence pathway	Effect	SE	LLCI	ULCI
Total effect	Mental health⇒sport participation attitudes	0.106	0.013	0.081	0.131
Direct effect	Mental health⇒sport participation attitudes	0.07	0.013	0.046	0.095
Total indirect effect	Mental health⇒sport participation attitudes	0.035	0.005	0.026	0.045
Indirect effect	Mental health⇒exercise self-efficacy⇒sport participation attitudes	0.026	0.004	0.018	0.034
	Mental health⇒sport confidence⇒sport participation attitudes	0.007	0.002	0.003	0.012
	Mental health⇒exercise self-efficacy⇒sport confidence⇒sport participation attitudes	0.002	0.001	0.001	0.003

In summary, independent mediation effects and chain mediation effects of exercise self-efficacy and sport confidence were identified between mental health and adolescent sport participation attitudes, fully revealing the progressive psychological pathways through which mental health is associated with adolescent sport participation attitudes.

## 4 Discussion

### 4.1 The role of adolescent mental health in sport participation attitudes

This study confirms a significant positive association between adolescent mental health and sport participation attitudes. These results align with existing research, Sound mental health helps adolescents maintain stable emotions and cope with setbacks in sports activities more positively, which further fosters positive cognition of personal sports ability; Higher exercise self-efficacy reduces individuals’ tendency to avoid sports and improves active participation willingness, and eventually translates into positive attitudes toward sport participation (Guo et al., 2015; Keyes, 2005). From the perspective of social cognitive theory, a positive mental health can optimize self-regulation and behavioral execution, encouraging individuals to actively engage in sports activities; conversely, psychological distress may trigger negative suggestions and a defeatist attitude, inhibiting the intention to participate (Bandura and Wessels, 1997). In terms of effect size interpretation, the correlation coefficients between mental health and sport confidence and sport participation attitudes are classified as small effects according to conventional benchmarks in social and sport psychology (Cohen, 2013). This outcome is consistent with the characteristics of the adolescent sample. Adolescents’ sport participation attitudes are jointly influenced by family, peers, school climate, personal hobbies and physical fitness, so a single psychological indicator (mental health) cannot produce a strong linear correlation. Nevertheless, these statistically significant small correlations represent stable intrinsic psychological associations, demonstrating that mental health is a meaningful predictor of adolescents’ sport-related psychological states and participation tendencies. Comparative analysis of the literature indicates that this study’s conclusions are consistent with various studies on adolescents sports psychology. Yu et al. (2024) confirmed that sport confidence occupies a proximal position within sport participation attitudes, offering greater explanatory power for participation behavior; additionally, Lochbaum et al. (2022) in their meta-analysis indicated that the strength of the association between sport confidence and sports behavior is significantly higher than that of distal psychological variables.

### 4.2 Exercise self-efficacy as a key mediator

Apart from direct effects, this study further found that exercise self-efficacy is a key mediator in the relationship between mental health and sport participation attitudes, with a stronger mediating effect than sport confidence. This stronger effect can be attributed to the distinct nature of the two constructs. Exercise self-efficacy, rooted in Bandura’s (2001) social cognitive theory, refers to an individual’s context-specific belief in their capability to successfully perform exercise-related tasks despite barriers (Beauchamp et al., 2019). It is inherently dynamic, task oriented, and sensitive to mastery experiences, vicarious learning, and physiological states—all of which are directly modifiable through sport participation. In contrast, sport confidence is often conceptualized as a more general and trait-like disposition, reflecting an overall belief in one’s ability to succeed in sport settings (Vealey, 1986). While confidence matters, it tends to be less responsive to short-term fluctuations in mental health and less actionable in intervention design. Specifically, when mental health deteriorates, self-efficacy, being more malleable and closely tied to perceived behavioral control, can more effectively transmit the negative impact of poor mental health onto reduced sport participation attitudes. Conversely, sport confidence, being relatively stable and global, may partially buffer but not fully capture the variability induced by mental health changes. Thus, from both a theoretical and practical standpoint, exercise self-efficacy serves as a more sensitive and proximally relevant mechanism linking mental health to sport participation attitudes, highlighting it as a prime target for psychosocial interventions.

Moreover, our study also found that exercise self-efficacy is positive associated with sport confidence, and further connects with sport participation attitudes through it. This finding is consistent with the hierarchical logic of self-efficacy theory, in which mental health first connects with the cognitive aspect of exercise self-efficacy, which then shapes the belief aspect of sport confidence, ultimately influencing sport participation attitudes. Specifically, exercise self-efficacy enhances adolescents’ positive perceptions of their exercise capabilities, reduces the fear of failure in sports, and thus increases the proactivity of sport participation attitudes, which is consistent with existing research findings (Feltz et al., 2008). Mechanistically, exercise self-efficacy operates through the pathway of capability belief — behavioral intention, where mental health enhances emotional stability and resilience to frustration, promoting positive exercise capability cognition; high self-efficacy reduces exercise avoidance and increases proactivity, ultimately transforming into positive participation attitudes (Hou et al., 2022; Yang et al., 2025). These results clarify that exercise self-efficacy, as a cognitive mediator, serves as a critical bridge in the transformation of distal psychological resources into sport participation attitudes.

### 4.3 The less direct but important role of sport confidence

While sport confidence only showed a modest mediation effect on the association between mental health and sport participation attitudes, its role is important in this dynamic through its association with exercise self-efficacy. On the one hand, mental health is positively related to sport participation attitudes via sport confidence—a proximal mediator. Based on Self-Determination Theory, positive mental health satisfies adolescents’ autonomy and competence needs, reduces failure anxiety, and builds self-confidence (Deci and Ryan, 2000). Confidence then activates a belief-reinforcement and behavior-activation pathway: it alleviates sports anxiety and strengthens behavioral approach tendencies, leading to positive attitudes. Our data confirm this robust mediating effect across groups, illustrating how distal psychological resources translate into participation behaviors through sport confidence. However, its role in this dynamic is less prominent, compared with exercise self-efficacy. On the other hand, sport confidence also transfer the effect of exercise self-efficacy to sport participation attitude. This finding, revealing the hierarchical internal mechanism of the conversion of distant psychological resources into participatory behavior. From the perspective of cognitive development and belief construction theories, mental health provides emotional stability and cognitive clarity for the assessment of athletic ability (Raglin, 2001); exercise self-efficacy is the belief in the controllability of abilities formed during exercise tasks (Hagger et al., 2001; Ryan and Dzewaltowski, 2002); and sport confidence represents a stable situational belief rooted in efficacy experiences. Together, these constructs form a psychological development sequence that progresses from cognition to belief, and from superficial to profound (Feltz, 1988; Jekauc et al., 2025). The data from this study precisely aligns with this pattern, fully presenting the chain transmission process that progresses from basic psychology to cognitive evaluation, then to stable beliefs, and ultimately drives participatory behavior (Jekauc et al., 2025).

Compared with independent mediation pathways, the chain mediation effect of exercise self-efficacy and sport confidence is relatively small. This is a typical feature of sequential chain mediation models (Hayes, 2017). When influences are transmitted through two consecutive psychological variables step by step, the effect magnitude naturally undergoes gradual attenuation during the transmission. Although the chain indirect effect is numerically small and belongs to a weak effect magnitude, it has irreplaceable theoretical, practical and clinical significance. Theoretically, this small but significant effect clearly verifies the hierarchical progressive relationship between cognitive belief (exercise self-efficacy) and emotional belief (sport confidence), and improves the theoretical framework of adolescent sport participation psychology. Practically, this finding explains why single intervention cannot rapidly change adolescents’ sport participation behaviors: the transformation from mental health to active sport participation is a gradual psychological development process. For school physical education and adolescent mental health education practitioners, this result indicates that we should adopt long-term, staged intervention strategies: starting with improving adolescents’ mental health, then cultivating exercise self-efficacy, and finally strengthening sport confidence, so as to continuously and subtly promote positive sport attitudes. Clinically, for adolescents with mild emotional and psychological problems, combined interventions targeting mental health and sport-related psychological qualities can serve as an effective auxiliary approach to increase their physical activity participation, which provides a new reference for adolescent psychological intervention and health management.

### 4.4 Limitations and future directions

This study has several limitations, which may provide directions for future research. First, a cross-sectional design was employed, revealing only the relationships between variables and preventing the strict inference of causal direction. Future studies should employ longitudinal tracking or experimental designs for further validation. Second, only age and gender were set as control variables in the statistical analyses. A series of important confounding variables closely related to adolescent sport participation, including socioeconomic status, parental education level, previous sports experience, weekly physical activity levels and school type, were not incorporated into the model. The omission of these factors may reduce the overall explanatory power of the research model and make it difficult to fully rule out alternative interpretations of the observed relationships, which is a notable limitation of this study. Thirdly, this study included only individual psychological variables, neglecting environmental and social factors such as family, school, peers, and cultural background. Adolescents’ sport participation attitudes result from the interaction between individual psychology and external environments. Future research could integrate multilevel variables to construct a more comprehensive individual-environment integration model. Fourthly, the core variables were measured using self-report scales, which can be influenced by social desirability, recall bias, and other subjective factors, with a single data source. Future studies could incorporate objective behavioral indicators and evaluations from teachers or parents as multisource data to enhance measurement validity. Finally, as the sample focused on adolescents, caution is advised when extrapolating conclusions to children or adult populations. Future research may conduct cross-age group comparative studies to examine the universality and developmental specificity of this chain mediation model.

## 5 Conclusion

This study investigates the internal mechanism through which Mental Health is associated with sport participation attitudes, uncovering its chain mediation effect through exercise self-efficacy and sport confidence. Findings clarifies the hierarchical psychological transmission mechanism of how mental health shows a significant correlation with sport participation attitudes among adolescents, delineating the progressive relationships of cognitive belief variables. It provides empirical evidence for enhancing mental health education, optimizing campus physical education, and intervening in sport participation attitudes, thereby contributing to the synergistic development of adolescents’ physical and mental health.

## Data Availability

The raw data supporting the conclusions of this article will be made available by the authors, without undue reservation.
