# Correction: How is mental health associated with teenagers' attitudes toward sport participation? — The chain mediating role of exercise self-efficacy and sport confidence

**DOI:** 10.3389/fpsyg.2026.1940329

**Published:** 2026-08-20

**Authors:** Jie Qiu, Mengfen Liu, Guiyun Zhang, Jiawei Chen, Haoyan Huang

**Affiliations:** 1 Hunan Mechanical and Electrical Polytechnic, Changsha, China; 2 Faculty of Educational Sciences, University of Helsinki, Helsinki, Finland

**Keywords:** exercise self-efficacy, mental health, sport confidence, sport participation attitudes, teenagers

The funder [Helsinki University Library] was erroneously omitted from the published article.

The correct funding statement appears below: The author(s) declared that financial support was received for this work and/or its publication. Open access was funded by Helsinki University Library.

The original version of this article has been updated.

